# Temporal Anticipation Based on Memory

**DOI:** 10.1162/jocn_a_01172

**Published:** 2017-08-04

**Authors:** André M. Cravo, Gustavo Rohenkohl, Karin Moreira Santos, Anna C. Nobre

**Affiliations:** 1 Federal University of ABC (UFABC), Santo André, Brazil; 2 Oxford Centre for Human Brain Activity, University of Oxford

## Abstract

The fundamental role that our long-term memories play in guiding perception is increasingly recognized, but the functional and neural mechanisms are just beginning to be explored. Although experimental approaches are being developed to investigate the influence of long-term memories on perception, these remain mostly static and neglect their temporal and dynamic nature. Here, we show that our long-term memories can guide attention proactively and dynamically based on learned temporal associations. Across two experiments, we found that detection and discrimination of targets appearing within previously learned contexts are enhanced when the timing of target appearance matches the learned temporal contingency. Neural markers of temporal preparation revealed that the learned temporal associations trigger specific temporal predictions. Our findings emphasize the ecological role that memories play in predicting and preparing perception of anticipated events, calling for revision of the usual conceptualization of contextual associative memory as a reflective and retroactive function.

## Introduction

Perception is increasingly recognized to be a highly proactive process resulting in a selective (re)construction of the external milieu that emphasizes items and attributes that may be adaptive in a given context. Goal-driven selective attention has provided a successful paradigm for investigating the sources and mechanisms of top–down modulation of signal processing within perceptual streams. Decades of research have yielded enormous progress in revealing how the locations and feature-related attributes of relevant events are prioritized and integrated along the sensory hierarchies (Fries, 2015; Reynolds & Chelazzi, 2004; Kastner & Ungerleider, 2000; Desimone & Ducan, 1995). These top–down biases were subsequently shown also to carry dynamic information about the estimated timing of relevant events—a phenomenon called temporal orienting of attention or, more generally, temporal expectation (Nobre & Rohenkohl, 2014). Trying to understand how temporal predictions of relevant events are extracted and can guide top–down control has become an active area of research, with promising inroads being made (Calderone, Lakatos, Butler, & Castellanos, 2014; Cravo, Rohenkohl, Wyart, & Nobre, 2013; Rohenkohl & Nobre, 2011; Lakatos, Karmos, Mehta, Ulbert, & Schroeder, 2008; Doherty, Rao, Mesulam, & Nobre, 2005; Vangkilde, Coull, & Bundesen, 2005).

As the attention field matures, scholars have returned to older hypothesized sources of top–down control of perception. In addition to current goals uploaded into short-term stores, our long-term memories have been proposed to guide perception from the earliest days of empirical psychology (von Helmholtz, 1867). Contemporary research using various types of tasks vindicates this classic notion (Goldfarb et al., 2016; Kasper, Grafton, Eckstein, & Giesbrecht, 2015; Giesbrecht, Sy, & Guerin, 2013; Zhao, Al-Aidroos, & Turk-Browne, 2013; Hutchinson & Turk-Browne, 2012; Stokes, Atherton, Patai, & Nobre, 2012; Kunar, Flusberg, & Wolfe, 2008; Summerfield, Lepsien, Gitelman, Mesulam, & Nobre, 2006; Chun, 2000). The tasks used, however, tend to focus on static aspects of learned contingencies, such as the location or identity of a target within an array or scene. In the current study, we asked whether our long-term memories can also carry temporal information that can guide perceptual analysis proactively and dynamically to enhance the processing of anticipated target attributes at the right moment in time. The research builds on recent discoveries of mechanisms for encoding sequential and temporal information within memory systems (Davachi & DuBrow, 2015; Eradath, Mogami, Wang, & Tanaka, 2015; Eichenbaum, 2013; MacDonald, Lepage, Eden, & Eichenbaum, 2011; Dragoi & Buzsaki, 2006).

We designed a novel memory-based temporal orienting task, based on previous work in the spatial domain (Stokes et al., 2012; Summerfield, Rao, Garside, & Nobre, 2011; Summerfield et al., 2006), to test for performance benefits conferred by learned temporal associations between target items and complex contexts. In the current study, participants learn that the target event occurs after a specific temporal interval within a given context. They subsequently perform a memory-based temporal orienting task in which they are asked to detect (Experiment 1) or discriminate (Experiment 2) the target appearance in the studied contexts.

## Methods

### Participants

Ten volunteers (three women, seven men; mean age = 19.4 years) participated in Experiment 1 (detection), and 18 (7 women, 11 men; mean age = 20.17 years) participated in Experiment 2 (discrimination). They all gave informed consent. All had normal or corrected vision and were free from psychological or neurological diseases according to self-report. The number of participants was based on comparable sample sizes in the literature (Stokes et al., 2012; Summerfield et al., 2006). The experimental protocol was approved by the research ethics committee of the Federal University of ABC and the central university research ethics committee of the University of Oxford.

### Apparatus

The stimuli were created on MATLAB v.7.10 (The MathWorks, Inc., Natick, MA) and presented using the Psychtoolbox v.3.0 package for MATLAB (Brainard & Vision, 1997). Images were displayed on a 21-in. CRT with a spatial resolution of 1024 × 768 pixels and a vertical refresh rate of 60 Hz, placed 100 cm in front of the participant. Responses were collected via a response box (DirectIN High SpeedButton/Empirisoft, New York, NY).

### Stimuli and Task

We conducted two similar experiments, in which participants learned new associations about the timing of a target event occurring within a scene and then performed an orienting task requiring detection (Experiment 1) or discrimination (Experiment 2) of the target event occurring within the learned context. In Experiment 2, EEG activity was recorded during the performance of the final, temporal orienting task requiring target discrimination. Each experiment consisted of three different tasks that took take place on the same day: a learning task, a memory task, and a temporal orienting task. Participants performed a session of the learning task, followed by a memory task. They then performed another session of the learning task and one more session of the memory task. Finally, they performed the temporal orienting task.

### Experiment 1: Detection

#### Learning Task

During the learning task, participants viewed 96 complex scenes repeated in random order over five blocks and learned the time for a target event to occur within each scene. Scene stimuli were similar to those used by previous studies (Stokes et al., 2012; Summerfield et al., 2006, 2011), consisting of photographs of different indoor or outdoor views. Scenes were prepared using MATLAB and subtended 22° × 17° of visual angle at a viewing distance of 100 cm. Although we considered using dynamic scenes, this would have conflated the timing of the target event with a sequence of spatial and/or feature-related changes that need not specifically rely on learning temporal intervals.

Each scene was associated with a target event being presented in a specific time and place that remained fixed throughout the whole learning session. The target event occurred between 5° and 7° of visual angle along both the lateral and longitudinal axes and was preceded by a placeholder presented at the exact same location. Participants were instructed to learn when the target event was presented within each scene. The interval and location of the target within each scene were randomized between participants. A briefly presented target thus occurred at a precise moment within a static scene. This arrangement was chosen over presenting a target within an evolving animated context (film) because it eliminates the possibility of learning relying only on associations between the occurrence of the target and a sequence of spatial or features within the dynamic context. By using the simpler approach, it was possible to isolate the effects of learning a purely temporal association.

Each trial started with the presentation of one of the scenes and a fixation cue in the center of the screen. After a period of 1.5 sec, a placeholder black bomb (1° × 1°) was presented in either the upper or lower quadrant of the right or left side of the scene. After an interval of either 800 or 2000 msec, the bomb changed its color to blue (go target, 80% of the trials) or red (no-go target, 20% of the trials). The type of target (go or no-go) was randomized over scenes, and participants were instructed that the same scene could have go or no-go targets in different blocks. Half of the images (48 scenes) were associated with each interval (short or long). Participants were instructed to respond as quickly as possible to go targets. If participants responded correctly and under 600 msec, a smoky cloud was presented, indicating that the response was correct. If participants did not respond to go targets within 600 msec or if they responded to no-go targets, an explosion image was presented. The order of scene presentation was randomized in each block. Participants performed three learning blocks in a row and then performed a memory task. They then completed two more learning blocks followed by another memory task.

#### Memory Task

During the memory task, participants viewed the same 96 naturalistic scenes repeated in random order. The scenes were presented on their own (no bombs appeared) and remained on the screen until participants responded. Their task was to indicate if the scene was associated with a short (800 msec) or long (2000 msec) interval during the learning task. Responses were made using index/middle fingers of the right hand. Memory tasks were performed after three blocks of the learning task and after the final block of the learning task.

#### Temporal Orienting Task

After completing five blocks of the learning task and two memory tasks, participants performed the temporal orienting task. The task was similar in structure to the learning task. Participants viewed the same 96 scenes, in which a bomb changed color after a short or long interval. In most of the trials (67%), the interval in the orienting task was the same as the learned interval in the learning task. The scene therefore triggered a valid memory cue for target timing. In the remaining trials (33%), the interval was switched, and the scene provided an invalid temporal memory cue. As before, participants were instructed to respond as quickly as possible to go targets and to withhold responding to no-go targets. The temporal orienting task consisted of three blocks, each with 96 scenes. In each block, a different subset of the scenes was selected to have an invalid memory cue. No feedback (smoky cloud or explosion) was given during this task.

### Experiment 2: Discrimination

The second experiment served as a replication and extension of Experiment 1, with EEG recordings made during the orienting task. The experiment contained the same three phases. The major differences were that, instead of using go/no-go targets, a change in bomb color (blue or green) required a discrimination response. Participants were instructed to press the right button when the bomb turned blue and the left button when it turned green (the mapping of color and response was counter-balanced across participants). Blue and green bombs were equiprobable and occurred arbitrarily for each scene. Participants were instructed that each scene was associated with the target event being presented in a specific time and place but that there was no association between the scene and the color of the bomb. Instead of performing five learning blocks as in Experiment 1, participants performed seven learning blocks. The memory task was performed after four blocks of learning and then after the final learning task block. The temporal orienting task was performed last.

### EEG Recording and Preprocessing

Continuous recording from 64 ActiCap electrodes (Brain Products, München, Germany) at 1000 Hz referenced to FCz (AFz ground) provided the EEG signal. The electrodes were positioned according to the International 10–10 system. Additional bipolar electrodes recorded the EOG. EOG electrodes were placed to the side of each eye (horizontal EOG) and above and below the right eye (vertical EOG). EEG was recorded using a QuickAmp amplifier and preprocessed using BrainVision Analyzer (Brain Products). Data were downsampled to 250 Hz and rereferenced to the averaged earlobes. To remove eye blink artifacts, filtered data (0.05–30 Hz) were subjected to independent component analysis. Eye-related components were identified through comparison of individual components with EOG channels and through visual inspection. Vertical eye activity was removed using independent component analysis.

For analyses of the contingent negative variation (CNV), epochs were segmented from 250 msec before scene onset until 800 msec after cue presentation. Epochs containing excessive noise or drift (±100 μV at any electrode) or eye artifacts (saccades) were rejected. Saccades were identified as large deflections (±50 μV) in the horizontal EOG electrodes. All data were subsequently checked by visual inspection. Data from four participants were removed because of excessive eye movements (two participants) or an excessive number of rejected trials (two participants). A small proportion of trials of the remaining participants were rejected (0.05 ± 0.01). We focused our analyses on short and long valid cues, with an average of around 90 clean epochs per condition.

## Results

### Learning Task

**Figure 1:**
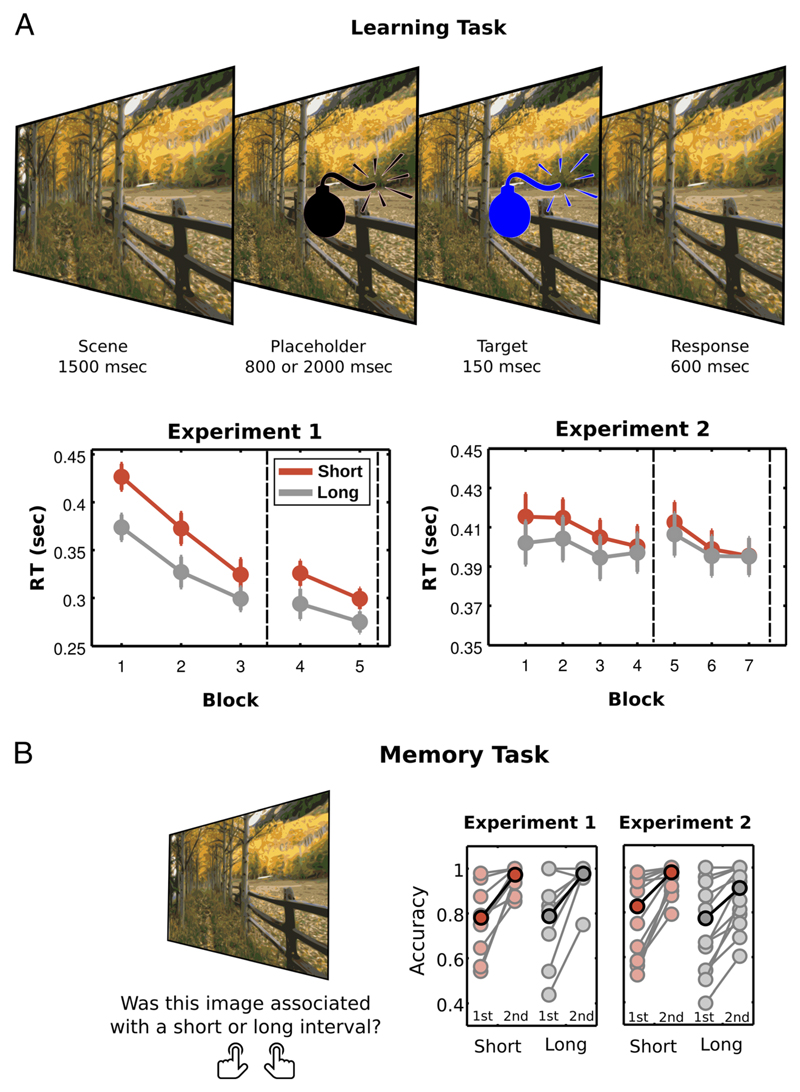
Learning and memory tasks. (A) During the learning task, participants viewed a complex scene and learned the temporal interval at which the target event occurred within that scene. After 1500 msec of the scene presentation, a placeholder (bomb) appeared. After an 800-msec (short) or 2000-msec (long) interval, the placeholder changed color. In Experiment 1, the target changed to blue in 80% of trials (go-target) or red in 20% of trials (no-go target). In Experiment 2, the target changed to blue or green in an equal proportion of trials. Participants had to detect the target (Experiment 1) or discriminate the color of the target (Experiment 2). In both tasks, participants’ RTs decreased as a function of Block, with a stronger effect for short intervals. The dashed lines represent when the memory task was performed in each experiment. All plots show mean and *SEM* across participants. (B) In the memory task, participants viewed each scene in isolation and indicated whether it was associated with a short or long interval. This task was performed by the participants halfway through the experimental session (first session) and at the end of the learning task (second session). Mean accuracies show how participants improved their performance over learning blocks, forming reliable explicit memories for the temporal associations between scenes and target presentation. All plots show mean accuracy across participants (darker colors) and raw data from all participants (lighter colors).

During the learning task (Figure 1A) of both experiments, participants viewed 96 scenes repeated in random order over five (Experiment 1) or seven (Experiment 2) blocks and learned the temporal interval at which the target event occurred within each scene. To quantify the improvement in performance in the learning tasks, RTs from the first and last blocks for short and long intervals were submitted to a 2 × 2 repeated-measures ANOVA, with factors Interval (Short × Long) and Block (First × Last).

In Experiment 1, participants had better performance at the end of the learning session for both short and long intervals (two-way Interval × Block ANOVA: main effect of Interval, *F*(1, 9) = 50.74, *p* < .001, η^2^_partial_ = 0.435; main effect of Block, *F*(1, 9) = 105.79, *p* < .001, η^2^_partial_ = 0.852; interaction, *F*(1, 9) = 7.56, *p* = .02, η^2^_partial_ = 0.063). However, learning was stronger for scenes with short intervals (*t*(9) = 2.75, *p* = .02, *d* = 0.869).

For Experiment 2, benefits in performance depended on the interval (two-way Interval × Block ANOVA: main effect of Interval, *F*(1, 13) = 9.25, *p* = .009, η^2^_partial_ = 0.016; no main effect of Block, *F*(1, 13) = 3.04, *p* = .11, η^2^_partial_ = 0.063; interaction, *F*(1, 13) = 5.09, *p* = .04, η^2^_partial_ = 0.007). Specifically, RTs improved only for short intervals (first vs. last blocks for short intervals, *t*(13) = 2.85, *p* = .014, *d* = 0.762, and long intervals, *t*(13) = 0.74, *p* = .47, *d* = 0.198). Thus, in both experiments, systematic decreases in RTs suggested that participants learned the temporal relationship between scenes and target intervals, with more pronounced learning for the short interval, as expected according to the hazard effect (Nobre & Rohenkohl, 2014; Cravo, Rohenkohl, Wyart, & Nobre, 2011).

### Memory Task

The memory task assessed whether participants formed an explicit memory for the temporal association within each scene (Figure 1B). The memory task was repeated midway through the learning task (after Block 3 in Experiment 1 and after Block 4 in Experiment 2) and after completion of the learning task. During the memory task, participants viewed each scene in isolation and indicated whether it was associated with a short or long interval. Mean accuracies for scenes with short and long intervals for the two blocks of the memory task were submitted to a repeated-measures ANOVA, with factors Interval (Short × Long) and Block (First × Last).

In both Experiments, there was an increase in accuracy as a function of learning (two-way Interval × Block ANOVA; Experiment 1: main effect of Block, *F*(1, 8) = 20.37, *p* = .002, η^2^_partial_ = 0.730; no main effect of Interval, *F*(1, 8) = 0.04, *p* = .84, η^2^_partial_ = 0.001; no interaction, *F*(1, 8) = 0.002, *p* = .97, η^2^_partial_ = 0; Experiment 2: main effect of Block, *F*(1, 13) = 23.02, *p* < .001, η^2^_partial_ = 0.352; no main effect of Interval, *F*(1, 13) = 3.74, *p* = .075, η^2^_partial_ = 0.065; no interaction, *F*(1, 13) = 0.269, *p* = .613, η^2^_partial_ = 0.001). The results showed that participants formed reliable explicit memories for the temporal associations between scenes and target presentation (Figure 1B).

### Orienting Task

The final orienting task probed whether the learned temporal associations influenced behavioral performance to expected targets. In most trials, the target occurred at the remembered interval (valid cue), whereas in the remaining trials, target occurred at the other interval, and the scene thus provided invalid temporal information (invalid cue).

**Figure 2:**
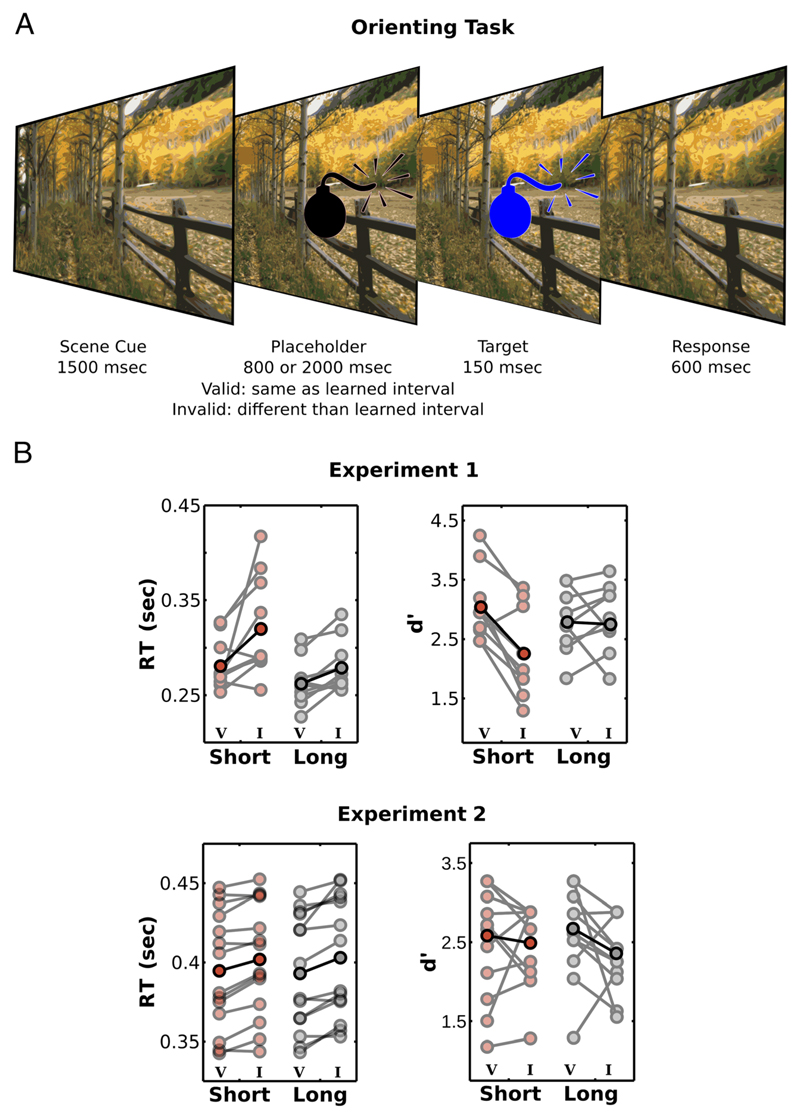
Temporal orienting task. (A) In the temporal orienting task, trial sequence was similar to the learning task; however, the interval when the target appeared matched that in the learning task in most trials (67% valid cues), whereas in the remaining trials (33% invalid cues), the target occurred at the other interval. (B) Performance was strongly influenced by long-term memory cues, and both RTs and perceptual sensitivity were better for valid (V) than invalid (I) scenes. All plots show mean across participants (darker colors) and raw data from all participants (lighter colors).

Mean RTs for correct responses were submitted to a repeated-measures ANOVA with Interval (Short × Long) and Cue (Valid × Invalid) as factors. As shown in Figure 2, performance was strongly influenced by long-term memory cues. In both experiments, RTs were shorter when targets were presented at the learned temporal interval (two-way Cue × Interval ANOVA; Experiment 1: main effect of Cue, *F*(1, 9) = 30.47, *p* < .001, η^2^_partial_ = 0.290; main effect of Interval, *F*(1, 9)= 10.14, *p* = .01, η^2^_partial_=0.254; no interaction, *F*(1, 9) = 2.3, *p* = .163, η^2^_partial_ = 0.020; Experiment 2: main effect of Cue, *F*(1, 13) = 20.14, *p* = .001, η^2^_partial_ = 0.029; no main effect of Interval, *F*(1, 13) = 0.42, *p* = .530, η^2^_partial_ = 0; no interaction, *F*(1, 13) = 0.023, *p* = .883, η^2^_partial_ = 0).

We further calculated *d*′ for each condition in the temporal orienting task. In Experiment 1, hits were considered as a correct response for a go target, whereas false alarms were considered when participants responded to a no-go target; *d*′ were submitted to a repeated-measures ANOVA with Interval (Short × Long) and Cue (Valid × Invalid) as factors. In Experiment 2, hits were calculated as correct response for green targets; and false alarms, as incorrect responses for blue targets; *d*′ were submitted to a repeated-measures ANOVA with Interval (Short × Long) and Cue (Valid × Invalid) as factors.

As can be seen in Figure 2, long-term memory also improved perceptual sensitivity for both detection (Experiment 1, two-way Cue × Interval ANOVA: main effect of Cue, *F*(1, 9) = 9.54, *p* = .013, η^2^_partial_ = 0.198; no main effect of Interval, *F*(1, 9) = 0.54, *p* = .481, η^2^_partial_ = 0.017; interaction, *F*(1, 9) = 9.72, *p* = .012, η^2^_partial_ = 0.081) and discrimination (Experiment 2, two-way Cue × Interval ANOVA: main effect of Cue, *F*(1, 13) = 7.33, *p* = .018, η^2^_partial_ = 0.066; no main effect of Interval, *F*(1, 13) = 0.05, *p* = .824, η^2^_partial_ = 0.001; no interaction, *F*(1, 13) = 0.70, *p* = .419, η^2^_partial_ = 0.010) tasks. For the detection task, perceptual sensitivity effects were restricted to the short interval (paired *t* test between valid and invalid cues for short intervals, *t*(9) = 4.64, *p* = .001, *d* = 1.467, and long intervals, *t*(9) = 0.20, *p* = .845, *d* = 0.063).

### CNV

In the orienting task of Experiment 2, analyses of the CNV focused in central midline electrodes (F1/Fz/F2/FC1/FC2) for scenes associated with short and long intervals during the learning task. A cluster-based analysis (Maris & Oostenveld, 2007) was applied to the whole period (from ‒200 msec before scene onset until 800 msec after the bomb was presented) to compare the CNV between conditions for the period between scene presentation and the first possible moment of the target. The nonparametric statistics were performed by calculating a permutation test in which experimental conditions were randomly intermixed within each participant and repeated 1000 times. The CNV for valid cues had higher (more negative) amplitudes for the period from 90 to 340 msec after cue presentation (cluster-stat = 202.05, cluster *p* = .002) and for the period from 390 to 800 msec after cue presentation (cluster-stat = 363.30, cluster *p* < .001).

**Figure 3:**
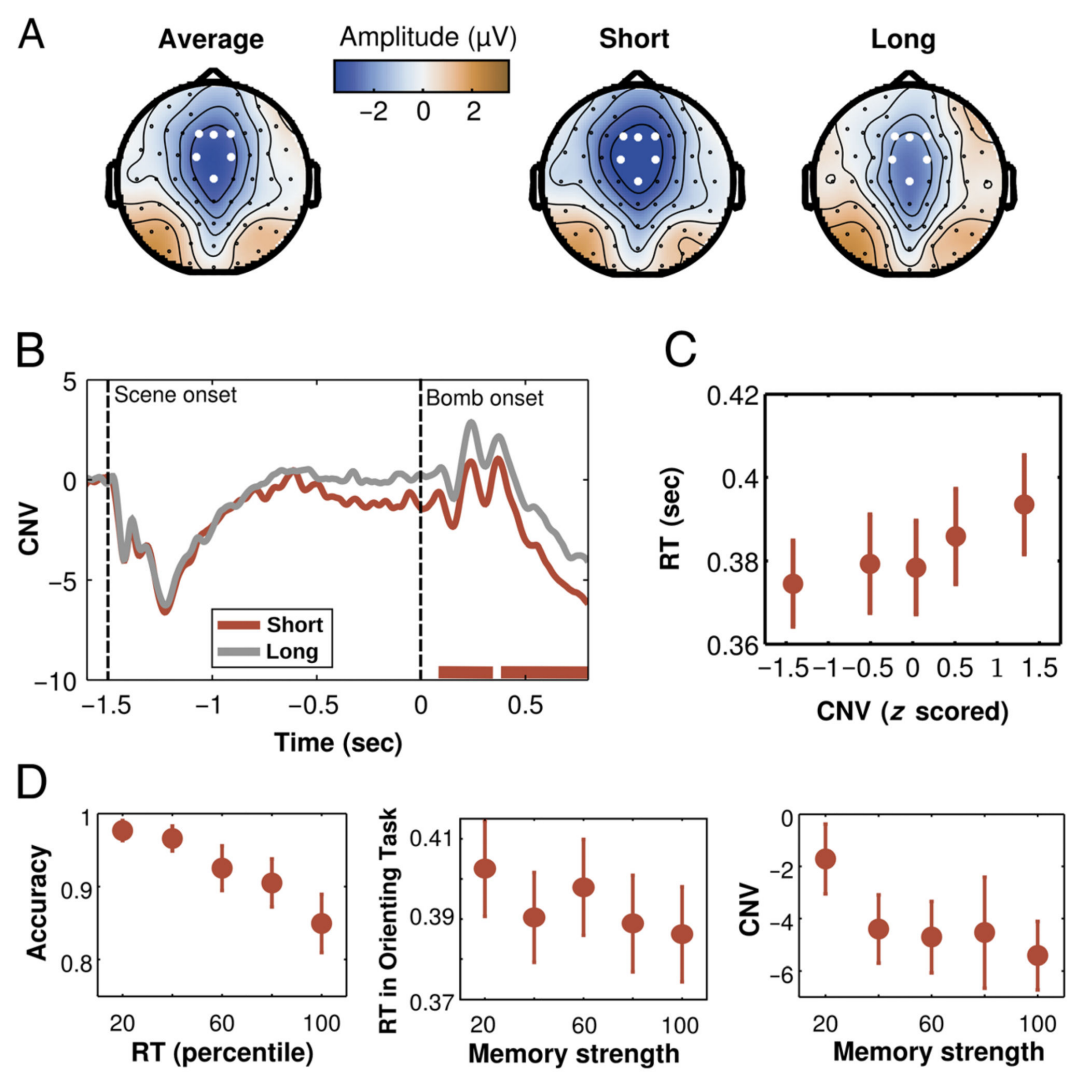
Electrophysiological results. (A) Topographies of the grand-averaged CNV and for the CNVs at the short foreperiod for scenes associated with short or long intervals. (B) The CNV recorded during the orienting task of Experiment 2 was strongly influenced by the temporal association in memory (red lines at the bottom represent the two temporal clusters where the CNV was larger for short than long temporal associations). (C) Larger CNV amplitudes were associated with shorter RTs (CNV values were binned into five equally sized bins for display purposes, although analyses were performed on raw data). (D, left) During the memory task, shorter RTs were associated with higher accuracy. Given this relation, RTs were used to create a memory strength index, which estimated the quality of the temporal association memory. Further analyses showed that stronger memories were associated with shorter RTs during the subsequent temporal orienting task (center) and with CNV amplitude (right). Memory strength was binned into five equally sized bins in the middle and right for display purposes, although analyses were performed on raw data. All plots show mean and *SEM* across participants.

To test whether the CNV reflected a stronger temporal anticipation, we investigated if there was a relation between CNV at the single-trial level and RTs. This analysis was performed in scenes associated with short intervals in the learning task and that were presented at the short interval in the temporal orienting task (short valid cues). The CNV activity for the second cluster (from 390 to 800 msec after cue onset) was averaged for each trial, *z* scored and separated into five bins (each with 20% of the data). The associated RT for each bin was calculated, and a nonparametric regression was calculated for each participant. At the group level, the Fisher-transformed estimated coefficients for the regression were compared with zero using a *t* test. We found that the amplitude of the CNV correlated significantly with RTs, indicating a functional relation between neural preparation and behavioral performance (*t*(13) = 2.69, *p* = .018, *d* = 0.719; Figure 3C).

### Memory Strength and Performance

An important property of learned temporal contextual associations is that their strength can vary. To estimate the strength of the temporal association memories, we used the RTs during the memory task.

In a first step, we investigated whether these RTs were correlated with response accuracy. For each participant, RTs for all scenes during the second memory task (after completion of the learning task) were separated into five bins, each containing 20% of the data. RTs shorter or longer than 2.5 *SD*s were removed before binning. For each bin, the mean accuracy was calculated. A nonparametric regression was performed separately for each participant. At the group level, the Fisher-transformed estimated coefficients were compared with zero using a paired *t* test. Participants formed stronger temporal memories for some scenes than for others as shown by the association between RT and accuracy during the memory test (*t* test on the estimated slopes, *t*(13) = −3.53, *p* = .004, *d* = 0.943; Figure 3D).

Given the strong association between RT and accuracy, we used these RTs as a memory strength index in two following analyses. In a first analysis, we investigated whether this index was associated with shorter RTs in the subsequent temporal orienting task. If participants had a stronger association between a given scene and its learned interval, then they should benefit more strongly from this association. We focused our analysis on (1) the first block of the temporal orienting task, (2) short valid trials, (3) trials in which participants gave correct responses in the temporal orienting task, and (4) scenes that participants judged correctly in the memory task. These restrictions were used to isolate as maximally as possible the effect of memory on performance.

For each trial in the temporal orienting task conforming to the abovementioned restrictions, the RT for that scene in the memory task was used as a predictor of the RT in the temporal orienting task. The memory strength index was calculated as the percentage of RTs that were longer than each individual RT. For example, for the shortest RT, all other RTs were longer, resulting in a memory strength index of 100. A nonparametric regression was performed with the RT in the temporal orienting task as the dependent variable and with the memory strength index as the predictor. At the group level, the Fisher-transformed estimated coefficients were compared with zero using a paired *t* test. As can be seen in Figure 3, memory strength was predictive of behavioral performance benefits (*t* test on the estimated slopes, *t*(13) = –2.71, *p* = .018, *d* = 0.723).

A similar analysis was performed to test whether this index was also related to the CNV. The same restrictions were used, and the memory strength index was calculated in a similar way. The CNV was measured in the same electrodes as previously mentioned and in the period of the second significant cluster (390–800 msec). A nonparametric regression was performed with the CNV as the dependent variable and with the memory strength index as the predictor. At the group level, the Fisher-transformed estimated coefficients were compared with zero using a paired *t* test. Similar to behavioral performance, memory strength was also predictive of the CNV amplitude (*t* test on the estimated slopes, *t*(13) = –2.33, *p* = .037, *d* = 0.620).

## Discussion

Across two experiments, we found that participants were able to learn temporal associations between target items and complex contexts. This learning was beneficial in the orienting task, with participants responding faster and more accurately for scenes tested at the learnt interval. Our findings suggest that long-term memories can guide our perception and behavior dynamically, utilizing stored temporal associations of specific intervals to prepare neural activity for relevant upcoming events.

Our results contribute crucial insights to the understanding of the influence of timing in contextual long-term memory. The relationship between timing and long-term memory is attracting increasing interest. Most studies so far have considered how the temporal order of events is encoded (Ezzyat & Davachi, 2014; Dragoi & Buzsaki, 2006) or how temporal proximity and regularity can modulate retrieval (Schapiro, Kustner, & Turk-Browne, 2012; Schwartz, Howard, Jing, & Kahana, 2005). In our studies, it becomes clear that precise temporal intervals, and not only the order of events, can be learned. Furthermore, these stored temporal associations are projected dynamically to anticipate relevant items at just the right moment to optimize performance.

Previous studies in long-term memory and attention, using a similar task, have shown that learning spatial locations of events can improve perceptual sensitivity and RTs (Stokes et al., 2012; Summerfield et al., 2006, 2011). In these tasks, it has been suggested that the effects of allocating attention based on long-term memory or on a symbolic cue might share similar anticipatory brain states, as alpha desynchronization.

Similarly, in our results, we found that long-term memory modulated an electrophysiological marker consistently found in temporal attention studies, the CNV. Targets that appeared at the learnt moments presented CNVs with higher amplitude and were judged faster and more accurately. Importantly, how well a memory was stored influenced not only the benefit in performance but also CNV amplitude. The CNV has been traditionally linked to temporal expectation (Cravo et al., 2011; Praamstra, Kourtis, Kwok, & Oostenveld, 2006; Los & Heslenfeld, 2005; Pfeuty, Ragot, & Pouthas, 2005; Nobre, 2001). Similar to studies that investigate the CNV in tasks with voluntary and automatic deployment of temporal attention, we found that its amplitude and time course were strongly related to the moment of target presentation. Once again, the effects of long-term memory on performance seem to mimetize the neural correlates of the voluntary deployment of attention.

Combined with our previous findings, our results emphasize the ecological role that memories play not only in storing information but also in predicting and preparing perception. They cast long-term memories in a new light. Rather than emphasizing their reflective and retroactive role of reconstituting, or remembering past events, they highlight the proactive role they play in predicting and preparing perception dynamically by pre-membering anticipated events.

The findings open new lines of investigation into the mechanisms through which mnemonic temporal associations guide perception. A fuller understanding of human perception will require understanding of dynamic regulation by both top–down signals from long-term memories and short-term biases related to current goals and expectations.
